# Health-related quality of life is impaired in bleeding disorders of unknown cause: results from the Vienna Bleeding Biobank

**DOI:** 10.1016/j.rpth.2023.102176

**Published:** 2023-08-22

**Authors:** Dino Mehic, Stephan Schwarz, Ihor Shulym, Cihan Ay, Ingrid Pabinger, Johanna Gebhart

**Affiliations:** 1 Department of Medicine I, Clinical Division of Haematology and Haemostaseology, Medical University of Vienna, Vienna, Austria; 2 Centre of Physiology and Pharmacology, Institute of Vascular Biology and Thrombosis Research, Medical University of Vienna, Vienna, Austria; 3 IT-Systems and Communications, Medical University of Vienna, Vienna, Austria

**Keywords:** BDUC, mental health, physical health, platelet function defects, quality of life, von Willebrand diseases

## Abstract

**Background:**

Bleeding disorder of unknown cause (BDUC) is a diagnosis of exclusion after extensive investigation of coagulation and platelet function and is commonly seen among patients with mild-to-moderate bleeding disorders. Despite increasing awareness among treating physicians, little is known about the health-related quality of life (HrQoL) in BDUC.

**Objectives:**

To investigate HrQoL in patients with BDUC in comparison to the general population and patients diagnosed with other established bleeding disorders.

**Methods:**

Patients with mild-to-moderate bleeding disorders from the Vienna Bleeding Biobank, a prospective cohort study, were contacted via mail and phone to complete the 36-Item Health Survey Questionnaire form.

**Results:**

In total, 333/657 (50.7%) patients completed the 36-Item Health Survey Questionnaire. Patients with BDUC (*n* = 207, 62%) had significantly impaired HrQoL both in physical (47.8 vs 49.2) and mental health parameters (42.9 vs 51.0) compared to the general population (*n* = 2914, 56% females), which remained after adjustment for sex and age in multivariable linear regression. The impairment in HrQoL, compared to patients with von Willebrand disease, platelet function defects, or mild clotting factor deficiencies, did not prevail after adjustment for age and sex. In patients with BDUC, age and the presence of at least 1 comorbidity were associated with impaired physical health but not sex or bleeding severity. Of all analyzed bleeding symptoms, only joint bleeding was associated with impaired physical health and gastrointestinal bleeding with mental health in BDUC.

**Conclusion:**

The impairments in HrQoL in patients with BDUC emphasize the burden of BDUC on mental and physical well-being, encouraging early recognition and better counseling of patients with BDUC.

## Essentials

• Knowledge about the quality of life in bleeding disorder of unknown cause (BDUC) is scarce.
• Quality of life was assessed in patients from the Vienna Bleeding Biobank.
• Patients with BDUC had reduced health-related quality of life compared to the general population.
• Impairments in quality of life emphasize the burden on mental and physical well-being in BDUC.

## Graphical abstract

**Figure:**
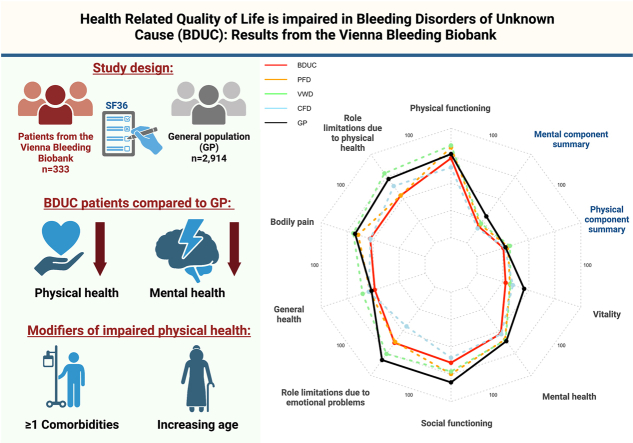

## 1 Introduction

Patients with mild-to-moderate bleeding disorders (MBD) suffer from symptoms like easy bruising, epistaxis, or mucocutaneous bleeding [1]. In most patients with MBD, no diagnosis can be established despite extensive and repeated laboratory investigations of plasmatic coagulation and platelet function (bleeding disorders of unknown cause, BDUC) [2]. Patients with BDUC do not differ from patients with a definite diagnosis of MBD, such as mild platelet function defects (PFD), von Willebrand disease (VWD), or mild coagulation factor deficiencies (CFD), with regards to their bleeding phenotype and bleeding severity [3,4]. Nevertheless, there is still a lack of awareness on BDUC among many treating physicians despite a lifelong psychological strain in some patients and potentially life-threatening bleeding during hemostatic challenges such as surgery or childbirth [5]. Many hemophilia centers do not register these patients in existing databases, albeit in some countries, the awareness has been increasing lately [5], and BDUC has been recognized as a distinct bleeding disorder [6].

Repeated bleeding events in patients with BDUC during their lifetime, as shown by our group [7], could potentially have a significant impact on patients’ mental and physical health. Previous studies have demonstrated that patients with both coagulation disorders, such as hemophilia or defects in primary hemostasis, including VWD and PFD, have impaired health-related quality of life (HrQoL) compared to the healthy population [[8], [9], [10], [11], [12]]. For patients with VWD, an association of the bleeding severity with HrQoL was also identified [13]. Despite the very high frequency of patients with BDUC among patients with MBD, large studies on the HrQoL of patients with BDUC have not yet been performed [10].

HrQoL can be studied by the short-form 36-Item Health Survey Questionnaire (SF-36), which is a tool to analyze self-reported aspects of life quality, quantifying patient-perceived well-being and functioning in terms of physical, emotional, mental, and social components and allowing comparison between distinct clinical conditions and with the healthy population [14,15].

In this study, we aimed to assess the HrQoL in a large cohort of patients from the Vienna Bleeding Biobank (VIBB), a single-center cohort study on patients with MBD. We compared parameters of HrQoL between patients with BDUC and those with VWD, PFD, or CFD, as well as the general population (GP). Furthermore, we investigated the association of clinical parameters and the bleeding phenotype with patients’ HrQoL.

## 2 Methods

### 2.1 Patients and study design

The VIBB is a single-center cohort study, established in 2009 and ongoing, including patients aged ≥16 years with a mild-to-moderate bleeding tendency. Detailed inclusion and exclusion criteria have been described previously and can be found in the Supplementary file (Supplementary Table S1). The study was approved by the Ethics Committee of the Medical University Vienna (EC no. 603/2009) according to the Declaration of Helsinki, and all patients had to sign a written informed consent form.

Based on a broad hemostatic laboratory assessment (Supplementary Table S2), patients were categorized as BDUC or obtained an established diagnosis of MBD (Supplementary Table S3). The detailed diagnostic work-up for VWD, PFD, and CFD in our study can be found in Supplementary Figure. For the diagnosis of VWD, both von Willebrand factor (VWF):antigen and VWF:ristocetin cofactor were measured, and since July 2015, VWF:glycoprotein (GP)1bM and VWF:collagen-binding were additionally assessed. Following diagnostic guidelines, pathologic VWF levels were defined as VWF:antigen and/or VWF activity (VWF:ristocetin cofactor and/or VWF:GP1bM) ≤50 International Units (IU)/dL [16].

Upon study inclusion, 2 standardized questionnaires were performed to quantify the bleeding severity: the Vicenza Bleeding Score (BS) and, since 2013, the bleeding assessment tool of the International Society on Thrombosis and Haemostasis (ISTH-BAT) [17,18]. Briefly, the applied standardized bleeding assessment tools record different bleeding symptoms, such as epistaxis, hematoma/easy bruising, small wound bleeding, oral mucosal and/or gingival bleeding, gastrointestinal bleeding, postpartum bleeding, muscle and/or joint bleeding, bleeding after tooth extraction, postsurgical bleeding, and menorrhagia and other bleedings in their most severe occurrence. In total, the Vicenza BS ranges from 0 (no symptoms) to 33 (all symptoms require medical intervention) points and the ISTH-BAT from 0 to 56. Cutoffs for pathologic bleeding are defined as ≥3 points for males and ≥5 points for females for the Vicenza BS, and ≥4 points for males and ≥6 points for females in the ISTH-BAT. All structured interviews on the medical history and bleeding severity were performed by trained health personnel upon study inclusion.

### 2.2 Health-related quality of life 36-Item Short-Form Survey

The SF-36 questionnaire (version 1) was sent out via mail to all patients who were included in the VIBB before May 2021. Patients who did not return the questionnaire were contacted via phone and asked to fill in an online form. All patients previously included in the VIBB study were contacted at the same time, while data on medical history and bleeding severity were recorded at study inclusion and thus at a different time point. The median (min-max) time between assessment of BS at study inclusion and time of SF-36 assessment was 71.1 (11.8-136.4) months.

The SF-36 records 36 items on 8 different dimensions: physical functioning (PF), role limitations due to physical health (RP), bodily pain (BP), general health (GH), vitality, social functioning (SF), role limitations due to emotional problems (RE), and mental health (MH) [15,19]. The acquired data of the SF-36 are transformed on a scale of 0 (worst health) to 100 (best health) for each dimension. Further, an overall physical component summary (PCS) and a mental component summary (MCS) are calculated based on the dimensions and predefined normative data of the SF-36. Normative data for the GP were obtained from a German health status survey [20].

### 2.3 Statistical analysis

Statistical analysis was performed with the Statistical Package for Social Sciences (SPSS IBM, version 26.0) and the free open-source software GNU R version 4.2.2. The difference in HrQoL dimensions in BDUC (compared with GP or other MBD diagnoses) was assessed in a multivariable linear regression model (adjusted for age and sex). For the correlation of the HrQoL score with BSs, Spearman correlation was performed. All *P* values are results of 2-sided tests, and *P* values <.05 were considered statistically significant.

## 3 Results

### 3.1 Patients’ characteristics

**Figure 1:**
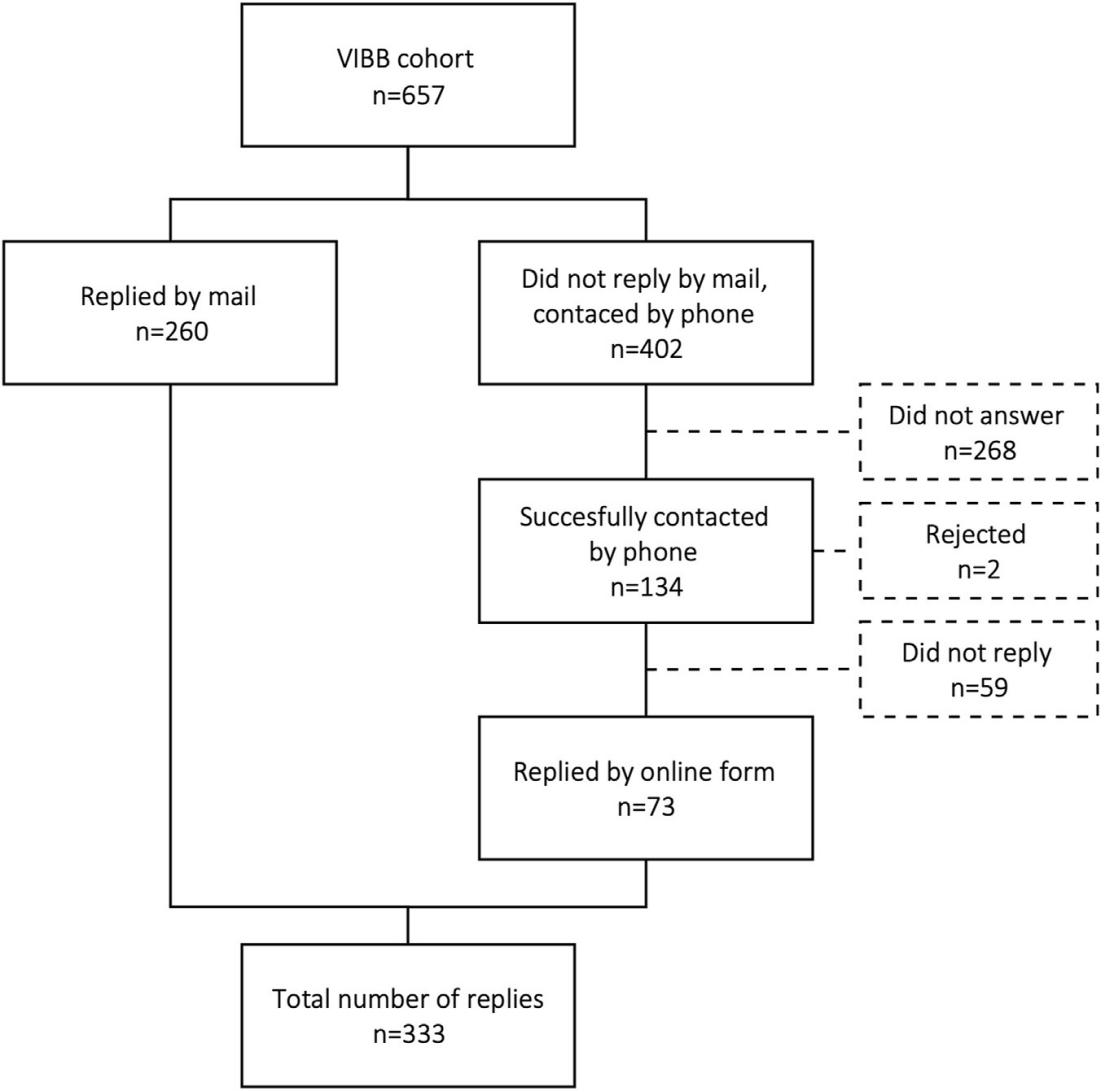
STROBE diagram of all included patients.

In total, 657 patients with MBD or BDUC were contacted to fill out the SF-36 questionnaire. Of these, 333 (50.7%) completed the SF-36 either online or sent the form by postal mail (Figure 1). Of the 333 analyzed patients, most were categorized as patients with BDUC (*n* = 207, 62.1%), while the remaining patients were diagnosed with VWD (*n* = 37, 11.1%), PFD (*n* = 74, 22.2%), or CFD (*n* = 12, 3.6%). Of the patients with VWD, most were of type 1 (*n* = 29, 78.4%), followed by type 2M (*n* = 4), 2A (*n* = 2), and 2B (*n* = 1) (in 1 patient, no distinction between 2A and 2B could be made). Patients with CFD were diagnosed with mild factor VIII deficiency (*n* = 9), FIX deficiency (*n* = 2), or FXI deficiency (*n* = 1). Three patients had other diagnoses (dys-/hypofibrinogenemia) and were not included in further analyses.

The detailed clinical characteristics are summarized in Table 1. Female sex was predominant in patients with BDUC, VWD, and PFD but not CFD. Patients with VWD were younger and had a higher blood group O rate than the other diagnostic groups. There was no difference in the prevalence of the most common comorbidities between the groups. Patients with VWD and CFD had a longer activated partial thromboplastin time, whereas there was no difference in platelet counts or hemoglobin levels between the diagnostic groups.

**Table 1:** Patients’ clinical and laboratory characteristics.

Characteristic	BDUC *N* = 207	PFD *N* = 74	VWD *N* = 37	CFD *N* = 12
	***N (%)***	***N (%)***	***N (%)***	***N (%)***
**Female sex**	174 (84.1)	55 (74.3)	31 (83.8)	5 (41.7)
**Positive family history**	81 (39.1)	24 (32.9)	22 (59.5)	3 (25.0)
**White race**	205 (99.0)	74 (100)	37 (100)	12 (100)
**Blood group O**	98 (47.6)	34 (46.6)	26 (70.3)	6 (50.0)
**Comorbidities ≥1**^a^	50 (24.3)	23 (31.5)	10 (27.0)	4 (33.3)
**Hypertension**	20 (9.7)	8 (10.8)	5 (13.5)	4 (33.3)
**Thyroid disease**	10 (4.8)	6 (8.1)	1 (2.7)	0 (0.0)
**Diabetes**	6 (2.9)	0 (0.0)	0 (0.0)	1 (8.3)
	***Median (IQR)***	***Median (IQR)***	***Median (IQR)***	***Median (IQR)***
**Age (y)**	43.0 (32.5, 55.0)	41.0 (28.2, 51.8)	32.0 (24.0, 41.0)	41.0 (24.8, 58.2)
**BMI**	23.4 (21.2, 26.5)	23.7 (20.3, 27.3)	22.7 (20.8, 25.7)	22.8 (22.1, 30.5)
**Vicenza BS**	6.0 (4.0, 8.0)	6.0 (4.0, 8.0)	6.0 (4.0, 9.0)	6.0 (5.0, 8.2)
**ISTH-BAT**^b^	6.0 (4.0, 9.0)	7.0 (4.8, 10.2)	6.0 (4.0, 9.0)	6.5 (4.0, 8.0)
**No. of bleeding manifestations**	3.00 (2.00, 5.00)	3.00 (2.00, 5.00)	4.00 (3.00, 5.00)	3.00 (2.50, 5.00)
**Platelets, G/OL**	239 (210, 278)	226 (186, 264)	238 (211, 279)	217 (200, 246)
**Hemoglobin, mg/dL**	13.60 (13.00, 14.20)	13.70 (12.93, 14.60)	13.40 (12.80, 14.50)	13.70 (12.70, 14.62)
**aPTT, s**	35.5 (33.5, 38.4)	36.2 (33.7, 39.2)	40.0 (36.6, 43.3)	45.9 (43.3, 52.5)
**PT, %**	97 (89, 104)	96 (89, 104)	91 (86, 97)	96 (90, 100)

aPTT, activated partial thromboplastin time; BDUC, bleeding disorder of unknown cause; BMI, body mass index; BS, bleeding score; CFD, clotting factor deficiency; ISTH-BAT; International Society on Thrombosis and Haemostasis; PFD, platelet function defect; PT, prothormbin time; VWD, von Willebrand disease.

a Includes hypertension, thyroid disease, diabetes, coronary disease, asthma, and gynecologic/urological disease.

b ISTH-BAT available for 144/207 (69.6%) patients with BDUC, 56/74 (75.7%) patients with PFD, 30/37 (81.1%) patients with VWD, and 8/12 (66.7%) patients with CFD.

The bleeding severity, as assessed by the Vicenza BS and ISTH-BAT, and the number of bleeding manifestations were similar between the groups.

### 3.2 Health-related quality of life in patients with bleeding disorder of unknown cause compared with healthy controls

**Figure 2:**
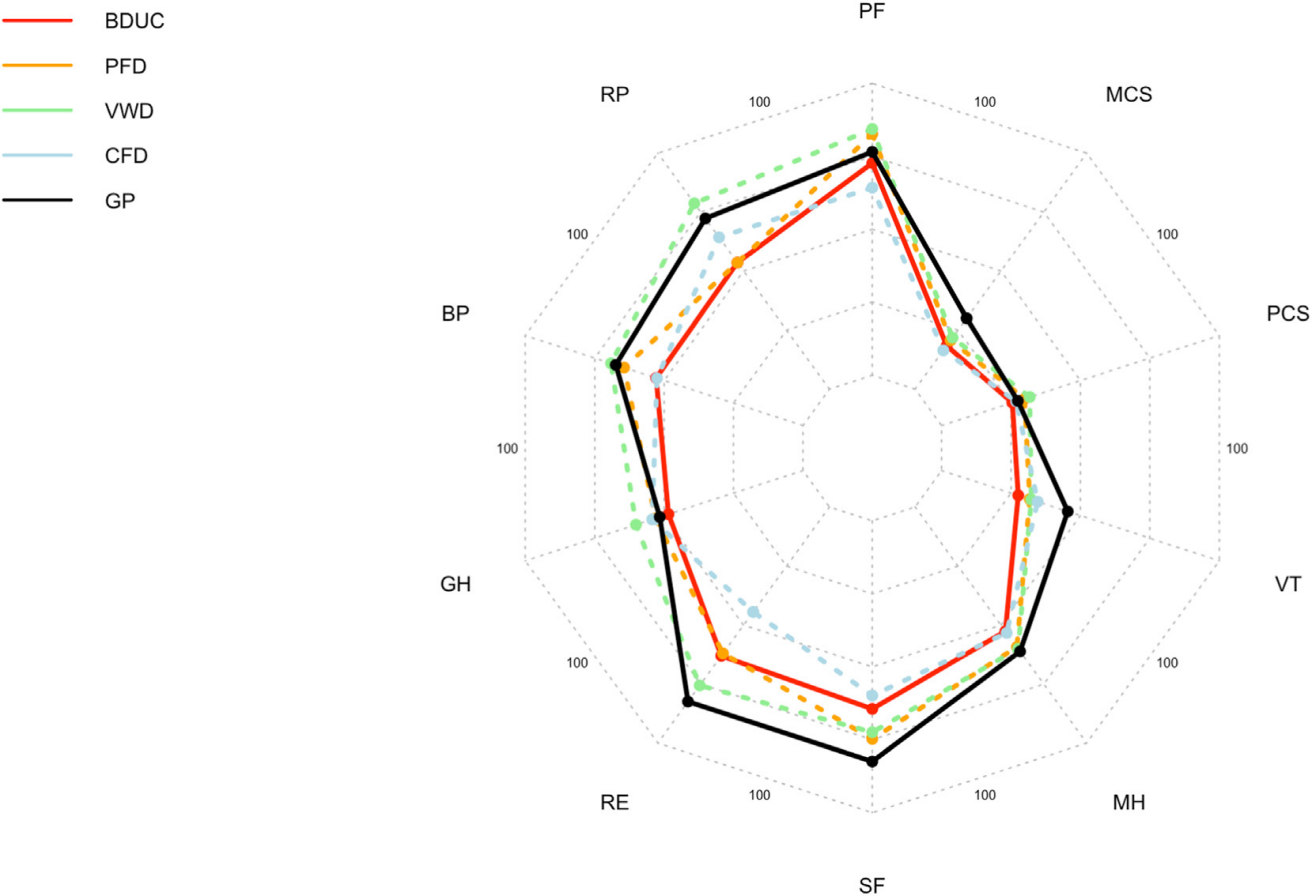
SF36 dimensions in MBD patients and the general population. BDUC, bleeding disorder of unknown cause; BP, bodily pain; CFD, clotting factor deficiency; GH, general health; GP, general population; MCS, mental component summary; MH, mental health; PCS, physical component summary; PF, physical functioning; PFD, platelet function defect; RE, role limitations due to emotional problems; RP, role limitations due to physical health; SF, social functioning; VT, vitality; VWD, von Willebrand disease.

Data on HrQoL in patients with BDUC was compared to normative data from the GP (*n* = 2914, 56% females) and had a mean (SD) age of 47.6 (17.8) years. In comparison to the GP, patients with BDUC exhibited significantly impaired HrQoL across all MH and most physical health parameters, as determined through the SF-36 questionnaire. These findings were obtained after adjusting for sex and age in a multivariable linear regression analysis (Table 2 and Figure 2). Of note, the nonsignificant impairment of PF, GH, and PCS in patients with BDUC became significant when accounting for age and sex in the adjusted multivariable linear regression model.

**Table 2:** 36-Item Health Survey Questionnaire dimensions in patients with BDUC compared with the GP.

SF-36 dimension	BDUC *N* = 207	GP *N* = 2914	Unadjusted		Adjusted for age and sex	
	**Mean (SD)**	**Mean (SD*)***	**MD**	**95% CI**	**MD**	**95% CI**
**Physical functioning**	80.9 (22.7)	83.6 (23.9)	-2.66	-6.02, 0.70	**-3.49**	**-6.44, -0.54**
**Role limitations due to physical health**	67.5 (41.6)	80.6 (34.5)	**-13.04**	**-17.99, -8.11**	**-13.17**	**-17.95, -8.40**
**Bodily pain**	67.0 (30.1)	77.2 (28.5)	**-10.14**	**-14.17, -6.10**	**-10.37**	**-14.23, -6.51**
**General health**	63.9 (19.7)	66.1 (21.2)	-2.16	-5.13, 0.81	**-3.24**	**-5.98, -0.49**
**Role limitations due to emotional problems**	74.1 (38.3)	87.7 (29.0)	**-13.67**	**-17.85, -9.48**	**-12.85**	**-17.04, -8.64**
**Social functioning**	75.1 (27.5)	87.7 (19.5)	**-12.61**	**-15.43, -9.77**	**-11.79**	**-14.62, -8.97**
**Mental health**	66.8 (18.5)	72.8 (17.4)	**-5.96**	**-8.42, -3.50**	**-4.48**	**-6.94, -2.03**
**Vitality**	49.3 (19.9)	61.8 (19.2)	**-12.48**	**-15.19, -9.76**	**-11.74**	**-14.40, -9.08**
**Physical component summary**	47.8 (11.0)	49.2 (10.9)	-1.42	-2.96, 0.13	**-1.95**	**-3.32, -0.57**
**Mental component summary**	42.9 (13.9)	51.0 (8.8)	**-8.05**	**-9.35, -6.75,**	**-7.27**	**-8.57, -5.97**

Missing values in the GP: PF 6, RP 14, BP 9, GH 1, RE 15, SF 3, MH 14, VT 26, PCS 53, and MCS 53.

BDUC, bleeding disorder of unknown cause; GP, general population; MD, mean difference.

The adjusted model revealed that BDUC diagnosis exhibited the greatest effect on the dimensions of RP, RE, SF, and vitality. The mean difference in the dimensions of HrQoL between the groups in unadjusted and adjusted analysis is shown in Table 2 to depict the changes in effect size after adjustment for age and sex.

### 3.3 Health-related quality of life in patients with bleeding disorder of unknown cause compared with other mild-to-moderate bleeding disorders

Next, we conducted a comparison of HrQoL dimensions between BDUC and other diagnoses of MBD using a linear regression model (Table 3 and Figure 2). Patients with BDUC yielded significant negative mean differences in PF, RP, and BP compared to PFD or VWD, lower SF values compared to PFD only, and lower GH compared with VWD. This resulted in a lower PCS in patients with BDUC than those with PFD or VWD. However, when adjusting for age and sex in a multivariable linear regression model, none of these mean differences remained statistically significant (Table 3). Interestingly, there was no significant difference in physical or MH parameters in patients with BDUC than CFD. The median group differences in the unadjusted and age- and sex-adjusted regression models are displayed in Table 3.

**Table 3:** 36-Item Health Survey Questionnaire dimensions in patients with BDUC compared with patients with platelet function defects, von Willebrand disease, and clotting factor deficiency.

SF-36 dimension	BDUC *N* = 207	PFD *N* = 74	Unadjusted		Adjusted for age and sex		VWD *N* = 37	Unadjusted		Adjusted for age and sex		CFD *N* = 12	Unadjusted		Adjusted for age and sex	
	**Mean (SD)**	**Mean (SD)**	**MD**	**95% CI**	**MD**	**95% CI**	**Mean (SD)**	**MD**	**95% CI**	**MD**	**95% CI**	**Mean (SD)**	**MD**	**95% CI**	**MD**	**95% CI**
**Physical functioning**	80.9 (22.7)	87.9 (19.9)	**-6.99**	**-12.85, -1.12**	-4.45	-9.94, 1.04	89.1 (18.5)	-8.14	**-15.91, -0.36**	-2.54	-9.72, 4.64	75.0 (27.1)	5.92	-7.50, 19.34	9.71	-2.96, 22.32
**Role limitations due to physical health**	67.5 (41.6)	79.7 (33.5)	**-12.22**	**-22.78, -1.65**	-9.02	-19.40, 1.35	85.1 (28.5)	-17.62	**-31.65, -3.60**	-12.00	-25.85, 1.84	75.0 (41.3)	-7.49	-31.80, 16.83	-0.98	-25.22, 23.25
**Bodily pain**	67.0 (30.1)	75.1 (29.1)	**-8.07**	**-16.02, -0.11**	-4.92	-12.61, 2.68,	78.4 (30.1)	-11.36	**-21.94, -0.79**	-6.55	-16.80, 3.77	66.8 (31.7)	0.18	-17.47, 17.83	5.9	-11.59, 23.40
**General health**	63.9 (19.7)	67.2 (20.7)	-3.34	-8.67, 1.99	-2.14	-7.45, 3.16	72.1 (21.8)	-8.24	**-15.29, -1.20**	-5.33	-12.33, 1.66,	68.0 (23.1)	-4.11	-15.76, 7.54	-2.88	-14.62, 8.86
**Role limitations due to emotional problems**	74.1 (38.3)	73.4 (40.5)	0.65	-9.72, 11.02	1.65	-8.16, 12.13	82.9 (33.9)	-8.81	-22.05, 4.43	-8.71	-22.26, 4.84,	61.1 (48.9)	12.96	-9.80, 35.72	15.85	-7.67, 39.36
**Social functioning**	75.1 (27.5)	82.3 (24.4)	**-7.20**	**-14.32, -0.08**	-6.22	-13.39, 0.94	80.7 (22.0)	-5.68	-15.08, 3.71	-4.17	-13.65, 5.26,	71.9 (30.7)	3.19	-12.99, 19.36	7.61	-8.98, 24.19
**Mental health**	66.8 (18.5)	71.5 (16.6)	-4.68	-9.49, 0.13	-4.26	-9.12, 0.59	72.0 (14.6)	-5.16	-11.50, 1.17	-4.98	-11.45, 1.48,	67.3 (23.8)	-0.50	-11.52, 10.52	0.93	-10.48, 12.34
**Vitality**	49.3 (19.9)	52.2 (19.6)	-2.89	-8.17, 2.40	-2.32	-7.62, 2.98	52.6 (20.6)	-3.29	-10.33, 3.75	-3.33	-10.52, 3.86,	54.2 (20.0)	-4.89	-16.55, 6.76	-3.39	-15.46, 8.69
**Physical component summary**	47.8 (11.0)	50.9 (9.8)	**-3.09**	**-5.94, -0.25**	-1.88	-4.55, 0.79	52.2 (11.1)	-4.46	**-8.32, -0.60**	-1.95	-5.58, 1.68,	48.8 (10.8)	-1.00	-5.41, 7.40	0.74	-5.39, 6.87,
**Mental component summary**	42.9 (13.9)		-1.53	-5.17, 2.11	-1.51	-5.16, 2.15	45.3 (11.5)	-2.39	-7.15, 2.38	-2.99	-7.84, 1.86,	41.5 (15.7)	1.40	-9.57, 6.78	2.4	-6.06, 10.87

BDUC, bleeding disorder of unknown cause; CFD, clotting factor deficiency; MD, mean difference; PFD, platelet function defect; SF-36, 36-Item Health Survey Questionnaire; VWD, von Willebrand disease.

### 3.4 Association of clinical parameters and bleeding severity with health-related quality of life in patients with bleeding disorder of unknown cause

In the association of clinical factors with SF-36 dimensions (Supplementary Table S4), age showed a moderate negative correlation with PF and weak negative correlations with RP, BP, GH, and PCS.

Female patients were more impaired in their PF, BP, and SF, while there were no overall differences in the PCS and MCS variables (Supplementary Table S5). Apart from RE, which was more impaired in patients with blood group non-O, there was no difference in the remaining SF-36 dimensions and component summaries with regard to blood group distribution (Supplementary Table S6).

In the correlation analysis, the Vicenza BS and the number of bleeding manifestations only showed weak to negligible negative correlations with the SF-36 dimensions (Supplementary Table S7) with weak negative correlations to PF, BP, GH, SF, and PCS, but not MH parameters. There was no relevant correlation with the ISTH BS.

In a multivariable linear regression model, only age and the presence of other comorbidities, but not sex or the bleeding severity (Vicenza BS and number of bleeding manifestations), were associated with impaired physical health, measured by the PCS. None of the studied characteristics were associated with MCS impairments (Table 4).

**Table 4:** Association of selected clinical parameters with health-related quality of life in patients with bleeding disorder of unknown cause.

Parameter	Physical component summary		Mental component summary	
	ß^a^	95% CI	ß^a^	95% CI
**Age (y)**	**-0.21**	**-0.31, -0.11**	0.13	-0.01, 0.27
**Female sex**	-2.7	-6.7, 1.3	-2.6	-8.0, 2.8
**Comorbidities ≥1**^b^	**-3.5**	**-6.9, -0.04**	-3.5	-8.2, 1.1
**Vicenza BS**	-0.27	-1.2, 0.71	-0.31	-1.6, 1.0
**No. of bleeding manifestations**	-0.21	-2.0, 1.6	-0.23	-2.7, 2.2

BS, bleeding score.

a Coefficient in multivariable linear regression.

b Includes hypertension, thyroid disease, diabetes, coronary disease, asthma, and gynecologic/ urological disease.

### 3.5 Association of bleeding symptoms with health-related quality of life in patients with bleeding disorder of unknown cause

We next performed a multivariable linear regression to study the association of distinct bleeding symptoms that are assessed with the Vicenza BS in the PCS and MCS. No clear association between the common bleeding manifestations in the PCS and MCS was seen. Only joint bleeding was associated with a reduced PCS and gastrointestinal bleedings with a reduced MCS (Table 5). Nevertheless, these bleeding manifestations were very rare, with 7 cases (3.3%) of joint bleedings and 25 cases (12.1%) of gastrointestinal bleedings.

**Table 5:** Association of bleeding symptoms with health-related quality of life in patients with bleeding disorder of unknown cause.

	Physical component summary		Mental component summary	
Bleeding symptom	ß^a^	95% CI	ß^a^	95% CI
**Easy bruising/ hematomas**	0.24	-3.4, 3.8	-1.7	-6.4, 2.9
**Small wounds bleedings**	-1.6	-4.9, 1.7	1.3	-2.9, 5.6
**Oral mucosal bleedings**	1.2	-2.3, 4.7	0.07	-4.4, 4.5
**Gastrointestinal bleedings**	-4.5	-9.2, 0.23	**-6.8**	**-12.8, -0.74**
**Bleeding after tooth extraction**	0.16	-2.1, 2.5	-0.89	-3.9, 2.1
**Postsurgery bleedings**	1.4	-1.2, 4.0	-1.1	-4.5, 2.2
**Heavy menstrual bleedings**	-2.5	-5.9, 0.93	-0.29	-4.7, 4.1
**Postpartum hemorrhage**	1.1	-0.15, 2.3	-0.06	-1.6, 1.5
**Muscle bleedings**	0.24	-12.5, 13.0	11	-5.8, 26.8
**Joint bleedings**	**-9.5**	**-17.9, -1.0**	9.6	-1.2, 20.5

a Coefficient in multivariable linear regression.

## 4 Discussion

Data on HrQoL have become increasingly important when assessing the health status of patient groups or the success of medical interventions, as physiological measures do not necessarily correlate with a patient’s well-being [21,22]. Despite growing evidence of hemostatic impairment [23,24], BDUC has only recently been acknowledged as a distinct bleeding disorder [5]. In this study, we demonstrate an impairment in HrQoL, both physical and mental, in patients with BDUC compared to the GP. Compared to patients with a diagnosis of bleeding disorders, patients with BDUC had similar physical and MH.

Although impaired HrQoL is a well-acknowledged burden of patients with bleeding disorders and has been reported in patients with VWD [25], PFD [12], or mild CFD [26,27], HrQoL in patients with BDUC has not yet been investigated. The disease burden is well reflected in the relevantly deteriorated MH in patients with BDUC compared to the GP, as shown in our study. The deteriorations in MH were not age- and sex-dependent and similar in patients with BDUC, PFD, and VWD, which emphasizes the overall association of bleeding disorders with patients’ MH [28,29]. In comparison to the GP, patients with BDUC also had significant deteriorations in all dimensions of physical health, which were independent of age and sex. In contrast, compared to patients with VWD and PFD, which were younger, patients with BDUC showed deteriorations in physical HrQoL only in unadjusted analysis, which did not prevail after adjustment for sex and age.

Increasing age has also been associated with reduced subjective health [30], as shown by the SF-36 questionnaire [31]. Also, our data showed an association between increasing age and impaired physical health in patients with BDUC. In general, impaired HrQoL in bleeding disorders has been attributed to recurrent bleeding events [25,27]. As age is a risk factor for bleeding, increased bleeding might explain the deteriorating effect of age on HrQoL in bleeding disorders [30]. Nevertheless, in our study, we did not identify an association between bleeding severity or the bleeding phenotype with HrQoL in patients with BDUC. This is in line with a previous Dutch study on patients with congenital PFD, which reported a reduced HrQoL based on the SF-36 questionnaire, which did not correlate with the BS [12,32,33]. In patients with moderate and severe VWD, De Wee et al. [13] showed a strong association of impaired HrQoL with the bleeding phenotype, whereas another study on patients with type 1 VWD only reported a trend toward lower physical health with an increasing BS [34]. Also, blood group O, associated with a more severe bleeding phenotype independently of VWF levels in BDUC and recurrent bleeding after hemostatic challenges [7,23], was not associated with HrQoL parameters in patients with BDUC in our study.

Our data show a similar deterioration of physical HrQoL in patients with BDUC and CFD in adjusted and unadjusted analyses. Impairments of SF-36-HrQoL have previously been observed in CFD, such as hemophilia and hemophilia carriers [26,27]. In persons with hemophilia, the number of joint bleedings [35] and the joint status assessed by different standardized scores were associated with reduced physical HrQoL [36,37]. Despite the mild bleeding phenotype in our patients, this is in line with our data on the deteriorating effect of joint bleeding on physical health in patients with BDUC.

The majority of our patients with BDUC were females [3]. Previous studies have shown that women with bleeding disorders are more severely impaired in their HrQoL than men due to women-specific bleeding complications [10,38]. We observed reduced physical and SF and a trend toward impaired physical, but not MH, in women with BDUC than in men. Nevertheless, neither sex nor women-specific bleeding manifestations like heavy menstrual bleeding or postpartum bleeding were associated with reduced HrQoL in patients with BDUC in multivariable linear regression analyses in our study.

The presence of at least one comorbidity was associated with impaired physical HrQoL in our study, which is not surprising and has been demonstrated previously [39]. Nevertheless, patients with BDUC from the VIBB are generally in a good health condition, with only a minority suffering from comorbidities such as diabetes or cardiovascular disease. Furthermore, our strict study design excluded patients with impaired liver or kidney function and malignancy, one of the biggest disease burdens in the GP [40].

Most studies on HrQoL in bleeding disorders focus on patients with a more severe bleeding phenotype [10,12,13]. Patients in the current analysis were selected from the VIBB, which included adult patients with a mild-to-moderate bleeding tendency in whom no previous in-depth investigation for a bleeding disorder has been performed, and our patients have a relatively mild bleeding phenotype. According to our data, patients with MBD have significant impairments in physical and mental HrQoL despite a relatively mild bleeding phenotype. Consequently, factors impacting the well-being of those patients need to be identified to target and reduce the patient burden.

The diagnostic work-up of patients with MBD requires a comprehensive diagnostic approach [1]. Nevertheless, numerous factors can impact hemostasis and VWF levels specifically, which we accounted for by excluding patients with acute phase reactions, pregnancy, acute bleeding, and severe comorbidities. In line with current recommendations, repeat testing was not systematically performed in our study, with the limitation of potentially underdiagnosing mild bleeding disorders like VWD. According to our systematic approach (Supplementary Figure), we have included patients in the corresponding diagnostic group in case of mild abnormalities, eg, in platelet function assessments. Through this process, we tried to best select a cohort of patients with BDUC, which served as the focal group in this study [3,[41], [42], [43], [44], [45], [46], [47], [48], [49]].

To the best of our knowledge, we are the first to present HrQoL data in patients with BDUC from an unbiased and well-characterized cohort of patients with MBD, patients which underlines the clinical relevance and burden of patients with a mild bleeding disorder with unknown underlying cause. However, limitations of this study include potential selection bias, as not all patients could be reached to fill out the SF-36 questionnaire; nevertheless, the diagnostic distribution was similar to the total cohort (Supplementary Table S8). Due to organizational reasons, we used version 1 of the SF-36, which is based on the same questions and answers as version 2 but lacks partial linguistical changes to be more understandable [50]. Furthermore, the bleeding questionnaires were recorded at study inclusion and, thus, before the assessment of HrQoL in these patients. The BSs reflect a patient’s bleeding history throughout a lifetime; nevertheless, severe bleeding episodes in the past might not affect HrQoL to the same extent as current bleedings, as discussed by Blaauwgeers et al. [12]. Furthermore, it cannot be excluded that other factors, such as pain, uncertainty, and psychosocial factors, may also play important roles in the multifactorial nature of quality of life. For the regression analyses, we only used the Vicenza BS, as the ISTH-BAT was not available for all patients (238/333, 71.5%); nevertheless, there was a strong correlation between the ISTH-BAT and Vicenza BS (r = .85, *P* < .001) [4,7]. Third, although all our laboratory investigations are based on current consensus and recommendations [1,6,16], we cannot exclude that a diagnosis of VWD or PFD might have been missed due to the complex diagnostic work-up in these patients.

Unfortunately, other aspects of HrQoL such as education, employment, social status, stress and anxiety or in general, patients’ perception of their health, were not assessed in this study, and would be interesting to investigate in further studies**.** Additionally, it would be valuable to longitudinally collect HrQoL data throughout the lifespan of patients with MBD to assess a potential impact of early diagnosis of bleeding disorders and enhanced counseling on patient outcomes. As mentioned, a majority of patients with MBD remain without a definite diagnosis and are categorized as BDUC. Advocating for awareness of BDUC among treating physicians could also improve the overall HrQoL in these patients.

In conclusion, our data show an impairment in physical and mental HrQoL in patients with BDUC compared to the GP. Health-related quality of life was equally deteriorated in BDUC compared to other bleeding disorders. Age and sex were partly associated with alterations of the HrQoL in patients with BDUC, whereas the bleeding phenotype was not. Our data emphasize the mental and physical burden of BDUC and underscore the importance of a better pathophysiological understanding of BDUC to provide personalized treatment, which would not only prevent bleeding complications but might also improve overall HrQoL in patients with BDUC.
