# Supplementary material for: Health-related quality of life is impaired in bleeding disorders of unknown cause: results from the Vienna Bleeding Biobank
Source: Res Pract Thromb Haemost. 2023 Aug 22;7(6):102176. doi: 10.1016/j.rpth.2023.102176 (PMC10502434; doi:10.1016/j.rpth.2023.102176)
Supplement: Supplementary material [file mmc1.docx]

**Supplementary data**

**Health Related Quality of Life is impaired in Bleeding Disorders of Unknown Cause: Results from the Vienna Bleeding Biobank**

Dino Mehic^1,2^, Stephan Schwarz^1^, Ihor Sholym^3^, Cihan Ay^1^, Ingrid Pabinger^1^, Johanna Gebhart^1^

^1^ Clinical Division of Haematology and Haemostaseology, Department of Medicine I, Medical University of Vienna, Vienna, Austria

^2^ Institute of Vascular Biology and Thrombosis Research, Centre of Physiology and Pharmacology, Medical University of Vienna, Vienna, Austria

^3^ IT-Systems and Communications, Medical University of Vienna, Vienna, Austria

**Correspondence:**

Johanna Gebhart, MD, PhD

Clinical Division of Hematology and Hemostaseology

Department of Medicine I, Medical University of Vienna

Waehringer Guertel 18-20, A-1090 Vienna, Austria

Phone number: +43 1 40400 44170

Fax: +43 1 40400 59980

E-Mail: [johanna.gebhart@meduniwien.ac.at](mailto:johanna.gebhart@meduniwien.ac.at)

**Supplementary Table 1** Inclusion and exclusion criteria of the VIBB study

| **Inclusion criteria** | **Exclusion criteria** |
| --- | --- |
| - Aged ≥ 16 years - Referred to the hemostasis outpatient clinic - Mild-to-moderate bleeding tendency - No diagnosis of a bleeding disorder | - Surgery or delivery within the last six weeks - Bacterial infection within the last two weeks - Active malignancy - Pregnancy - Thrombocytopenia (<100 x10^9^/L), - Intake of anticoagulants/antiplatelet-/anti-inflammatory drugs (last 5-10 days) - Current acute phase reaction - Continuous anticoagulant and/or antiplatelet therapy - Impaired liver function (prothrombin time <75 % of normal due to deficiency in vitamin K-dependent clotting factors) - Impaired kidney function (GFR <60 mL/min/1.73 m^2^) |

**Supplementary Table 2:** Laboratory tests performed at study inclusion

| **Global coagulation tests and coagulation factor tests** |
| --- |
| Activated partial thromboplastin time (aPTT), seconds |
| Prothrombin time (PT), % |
| Fibrinogen-Clauss, mg/dL |
| Factor VIII activity, % |
| Factor IX activity, % |
| Factor XIII activity, % |
| Factor V activity in patients with PT<75% and aPTT>41 seconds |
| Factor VII activity in patients with PT < 75% |
| Factor XI activity in patients with aPTT > 41 seconds |
| Factor II activity in patients with aPTT > 41 seconds |
| Factor X activity in patients with aPTT > 41 seconds |
| **Von Willebrand diagnostic** |
| Von Willebrand factor antigen (VWF:Ag),I U/dL |
| Von Willebrand factor ristocetin cofactor activity (VWF:RCo), IU/dL |
| Von Willebrand factor activity (VWF:GP1bM), IU/dL, since 07/2015 |
| VWF:RCo (or VWF:GPIbM)/ VWF:Ag ratio with <0.7 as cutoff for type 2 |
| Von Willebrand factor collagen-binding assay (VWF:CB), IU/dL, since 07/2015 |
| VWF multimeric analysis if VWF:RCo (or VWF:GPIbM)/ VWF:Ag ratio <0.7 |
| **Platelet function tests** |
| **Parameters of light transmission aggregometry (Born)** |
| ADP (5 µM), % |
| Arachidonic acid (1.6 mM) , % |
| Collagen (10 µg/ml), % |
| Epinephrine (5,5 µM), % |
| Ristocetin 1.2mg/ml), % |
| Ristocetin (0.6 mg/ml), % |

**Supplementary Table 3 -** Diagnostic criteria for established diagnoses

| **Diagnosis** | **Criteria for diagnosis** |
| --- | --- |
| Bleeding disorder of unknown cause (BDUC) | Normal results in all laboratory tests on plasmatic coagulation and platelet function |
| Von Willebrand disease (VWD) | VWF activity (VWF:RCo, since 2015 also VWF:GP1bM) and/or antigen levels ≤50 IU/dL |
| Platelet function defect (PFD) | Abnormal LTA aggregation curves upon stimulation with 1 or more agonists (including patients with possible and definite PFD) |
| Coagulation factor deficiency (CFD) | Factor VIII deficiency (FVIII ≤50%)  Factor IX deficiency (FIX ≤50%)  Factor XIII deficiency (FXIII ≤30 %)  Factor XI deficiency (FXI ≤60%)  Factor II deficiency (FII ≤ 20%)  Factor V deficiency (FV≤ 10 %)  Factor VII deficiency (FVII ≤ 20%)  Factor X deficiency (FX ≤ 20%) |
| Hypo-/dysfibrinogenemia | Quantitative deficiency or qualitative defect of fibrinogen |

*VWF:Ag, von Willebrand factor antigen; VWF:RCo, von Willebrand factor activity*

**Supplementary Table 4 Correlation of SF36 parameters in BDUC patients with age**

|  | **Age** |
| --- | --- |
| **Physical functioning** | -0.426*** |
| **Role limitations due to physical health** | -0.223** |
| **Bodily pain** | -0.252*** |
| **General health** | -0.220** |
| **Role limitations due to emotional problems** | -0.031 |
| **Social functioning** | -0.024 |
| **Mental health** | -0.009 |
| **Vitality** | 0.005 |
| **Physical component summary** | -0.373*** |
| **Mental component summary** | 0.107 |

Spearman correlation significance: *p<0.05, **p<0.01, ***p<0.001

**Supplementary Table 5 Comparison of SF36 dimensions in BDUC patients according to sex**

|  | Male  N = 33 | Female  N = 174 | **p** |
| --- | --- | --- | --- |
|  | mean (SD) | mean (SD) |  |
| **Physical functioning** | 87.9 (20.6) | 79.6 (22.9) | **0.022** |
| **Role limitations due to physical health** | 81.1 (31.9) | 64.9 (42.7) | 0.060 |
| **Bodily pain** | 79.0 (26.0) | 64.7 (30.3) | **0.027** |
| **General health** | 66.5 (18.8) | 63.4 (19.9) | 0.423 |
| **Role limitations due to emotional problems** | 81.8 (34.5) | 72.6 (38.9) | 0.194 |
| **Social functioning** | 85.2 (26.0) | 73.1 (27.4) | **0.004** |
| **Mental health** | 70.6 (18.3) | 66.1 (18.5) | 0.166 |
| **Vitality** | 53.6 (20.3) | 48.4 (19.8) | 0.312 |
| **Physical component summary** | 51.0 (8.9) | 47.2 (11.2) | 0.053 |
| **Mental component summary** | 45.9 (13.6) | 42.4 (13.9) | 0.155 |

**Supplementary Table 6 Comparison of SF36 dimensions in BDUC patients according to blood group O (vs. non-O)**

|  | BG non-O  N = 108 | BG O  N = 98 | **p** |
| --- | --- | --- | --- |
|  | mean (SD) | mean (SD) |  |
| **Physical functioning** | 80.0 (24.6) | 81.8 (20.5) | 0.886 |
| **Role limitations due to physical health** | 64.4 (43.3) | 70.7 (39.7) | 0.301 |
| **Bodily pain** | 66.0 (31.3) | 68.2 (29.0) | 0.541 |
| **General health** | 63.0 (20.9) | 65.2 (18.3) | 0.722 |
| **Role limitations due to emotional problems** | 68.8 (40.6) | 80.3 (34.8) | **0.021** |
| **Social functioning** | 72.3 (28.7) | 78.3 (25.9) | 0.145 |
| **Mental health** | 65.3 (20.3) | 68.7 (16.4) | 0.400 |
| **Vitality** | 48.1 (21.2) | 50.6 (18.5) | 0.384 |
| **Physical component summary** | 47.6 (11.7) | 47.9 (10.2) | 0.884 |
| **Mental component summary** | 41.4 (14.8) | 44.8 (12.5) | 0.128 |

BDUC, bleeding disorder of unknown cause; BG, blood group

**Supplementary Table 7 Correlation of SF-36 parameters in BDUC patients with bleeding scores and number of bleeding manifestations**

| **Characteristic** | **Vicenza BS** | **ISTH BAT** | **Number of bleeding manifestations** |
| --- | --- | --- | --- |
| **Physical functioning** | -0.199* | -0.098 | -0.223*** |
| **Role limitations due to physical health** | -0.160* | -0.067 | -0.147* |
| **Bodily pain** | -0.148* | -0.114 | -0.146* |
| **General health** | -0.191* | -0.129 | -0.215** |
| **Role limitations due to emotional problems** | -0.122 | -0.130 | -0.091 |
| **Social functioning** | -0.147 | -0.110 | -0.186** |
| **Mental health** | -0.063 | -0.087 | -0.096 |
| **Vitality** | -0.063 | -0.039 | -0.070 |
| **Physical component summary** | -0.178* | -0.087 | -0.174 |
| **Mental component summary** | -0.071 | -0.093 | -0.090 |

BS, bleeding score; ISTH BAT, ISTH bleeding assessment tool

Spearman correlation significance: *p<0.05, **p<0.01, ***p<0.001

**Supplementary Table 8: Diagnostic breakdown of the VIBB cohort and included patients int this study**

|  | Patients included in this study | All included patients included in the VIBB until |
| --- | --- | --- |
| BDUC | 207 (62.1%) | 423 (64.4%) |
| PFD | 74 (22.2%) | 137 (20.9%) |
| VWD | 37 (11.1%) | 70 (10.6%) |
| CFD | 12 (3.6%) | 23 (3.5%) |
| Other | 3 (0.9%) | 4 (0.6%) |
| **Total** | **333 (100%)** | **657 (100%)** |

**Supplementary Figure 1 Diagnostic work-up for MBD within the Vienna Bleeding Biobank**

**
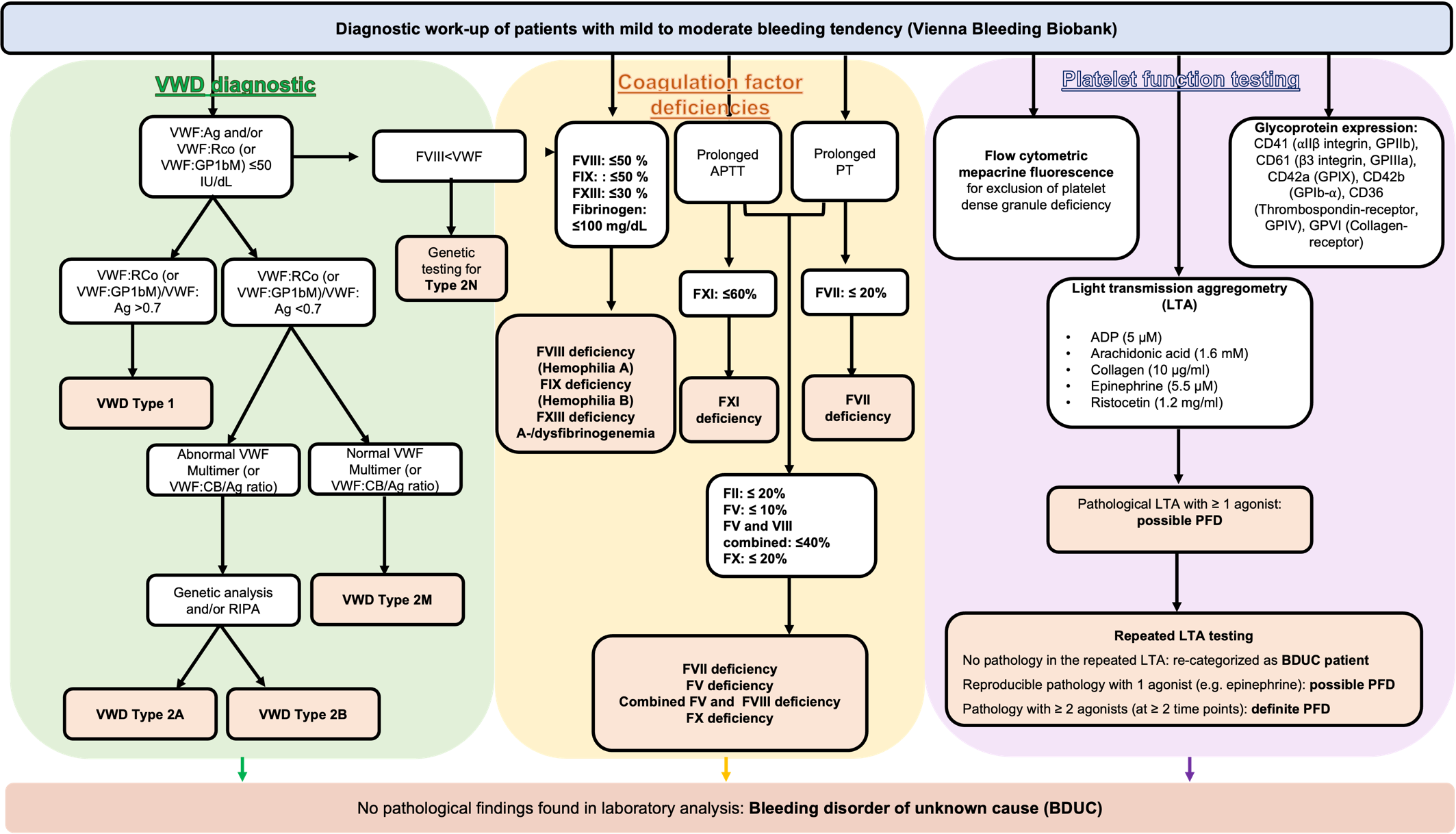
**

*MBD, mild to moderate bleeding disorders; VWD, von Willebrand disease; VWF, von Willebrand factor; RIPA, Ristocetin-induced platelet aggregation*
